# Vulvar migration of injected polyacrylamide hydrogel following breast augmentation: a case report and literature review

**DOI:** 10.1186/s12905-024-02998-0

**Published:** 2024-03-02

**Authors:** Junxian Wen, Zhijin Li, Yarong Chi, Bo Chen, Tao Hong, Zhifei Liu, Nanze Yu, Xiaojun Wang

**Affiliations:** 1 Department of Plastic Surgery, Peking Union Medical College Hospital, Chinese Academy of Medical Sciences, Peking Union Medical College, Beijing, China; 2 Peking Union Medical College (PUMC), PUMC & Chinese Academy of Medical Sciences, Beijing, China; 3 Department of Pathology, Peking Union Medical College Hospital, Chinese Academy of Medical Sciences, Peking Union Medical College, Beijing, China; 4 Department of General Surgery, Peking Union Medical College Hospital, Chinese Academy of Medical Sciences, Peking Union Medical College, Beijing, China; 5 Department of International Medical Service, Peking Union Medical College Hospital, Chinese Academy of Medical Sciences, Peking Union Medical College, Beijing, China

**Keywords:** Breast augmentation, Vulva, Polyacrylamide hydrogel, Migration, Case Report, Literature Review

## Abstract

**Background:**

Vulvar migration is a rare complication of filler injection for breast augmentation, generally presenting as repeated pain and fever. We will report a case of woman with polyacrylamide hydrogel breast injection develops vulvar abscess.

**Case presentation:**

A woman with a history of polyacrylamide hydrogel breast injection was noted to have vulvar abscess due to migration of filler materials. Filler removal surgery and vacuum sealing drainage was performed for this patient. The patient was discharged from the hospital with no further complications. After a review of pertinent literature, only four previous case reports are found. Local inflammatory response, infection, large volume injections, inframammary fold destruction, hematogenous or lymphatic migrate, trauma, gravity and external pressure could play essential parts in the migration of injected filler.

**Conclusion:**

Polyacrylamide hydrogel migration poses a worldwide challenge, necessitating personalized solutions. Our case study underscores the importance of comprehensive examinations for individuals with a history of filler breast injection when suspecting vulvar filler migration.

**Supplementary Information:**

The online version contains supplementary material available at 10.1186/s12905-024-02998-0.

## Background

Breast augmentation is a common and popular aesthetic surgical procedure, with nearly 300,000 surgeries are performed annually in America [1]. Although silicone gel or saline implants dominate the world market, the injection breast augmentation, a simple procedure with fast recovery, were once prevalent for female [2]. Paraffin, liquid silicone, polyacrylamide hydrogel(PAAG) and other materials have been injected into in breast tissue before [3]. As a non-resorbable sterile watery gel, PAAG has been applied in many clinical situations in China since 1997. PAAG was initially considered to be well tolerated by the breast and does not give rise to severe fibrosis, pain, or capsule shrinkage [4]. However, owing to the increased complications, PAAG injections have been shown to be potentially dangerous. The production and use of PAAG was eventually stopped by the China Food and Drug Administration (CFDA) in 2006 [5].

The complications following PAAG injections include inflammation, pain, subcutaneous nodules, infection and gel migration, which causes double injury to the body and mind in patients [4, 6, 7]. Migration is a common complication and can be easily diagnosed. However, distant migration is relatively rare, especially from breast to the vulva. To date, only few cases have been reported in the literature [4, 6, 8]. These patients only underwent vulvar mass excision or chose nonoperative treatment because of fear of more aggressive surgical plan. Therefore, lesions in their bodies were not be thoroughly debrided. We present a case of vulva migration following PAAG breast injection to raise awareness, discuss management and add new information to the current literature. Furthermore, the possible reasons of filler vulva migration will be discussed in details.

## Case presentation

A 35-year-old female patient underwent PAAG injection augmentation mammoplasty at a local private clinic in 2014. In March 2022, she developed a mass in the left of the vulva with pain, fever and hardness in the left lower quadrant of the abdomen. Two months antibiotic treatment according to the results of pus cultures and two removal surgeries of the filler material in the left vulva were performed but she still had sustained pain, fever, and restricted activity in lower limb. Eventually, she was referred to our institution for further proper management.

**Fig. 1:**
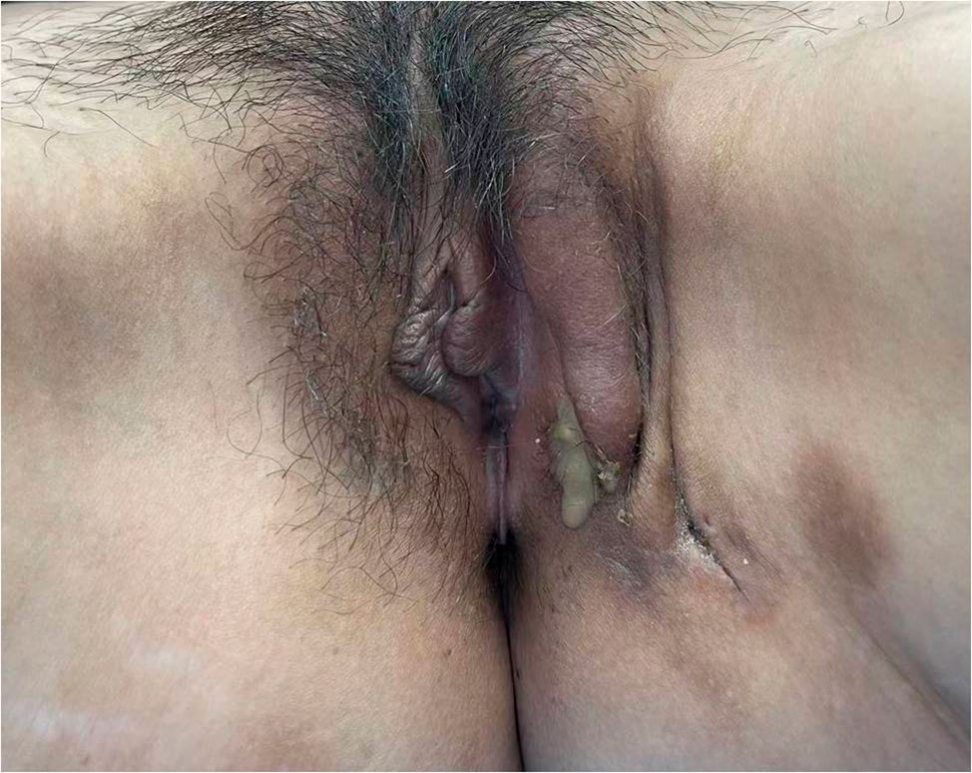
A 10*3 cm abscess on the left labia majora with yellow pus draining from a fistula under the vulvar skin

**Fig. 2:**
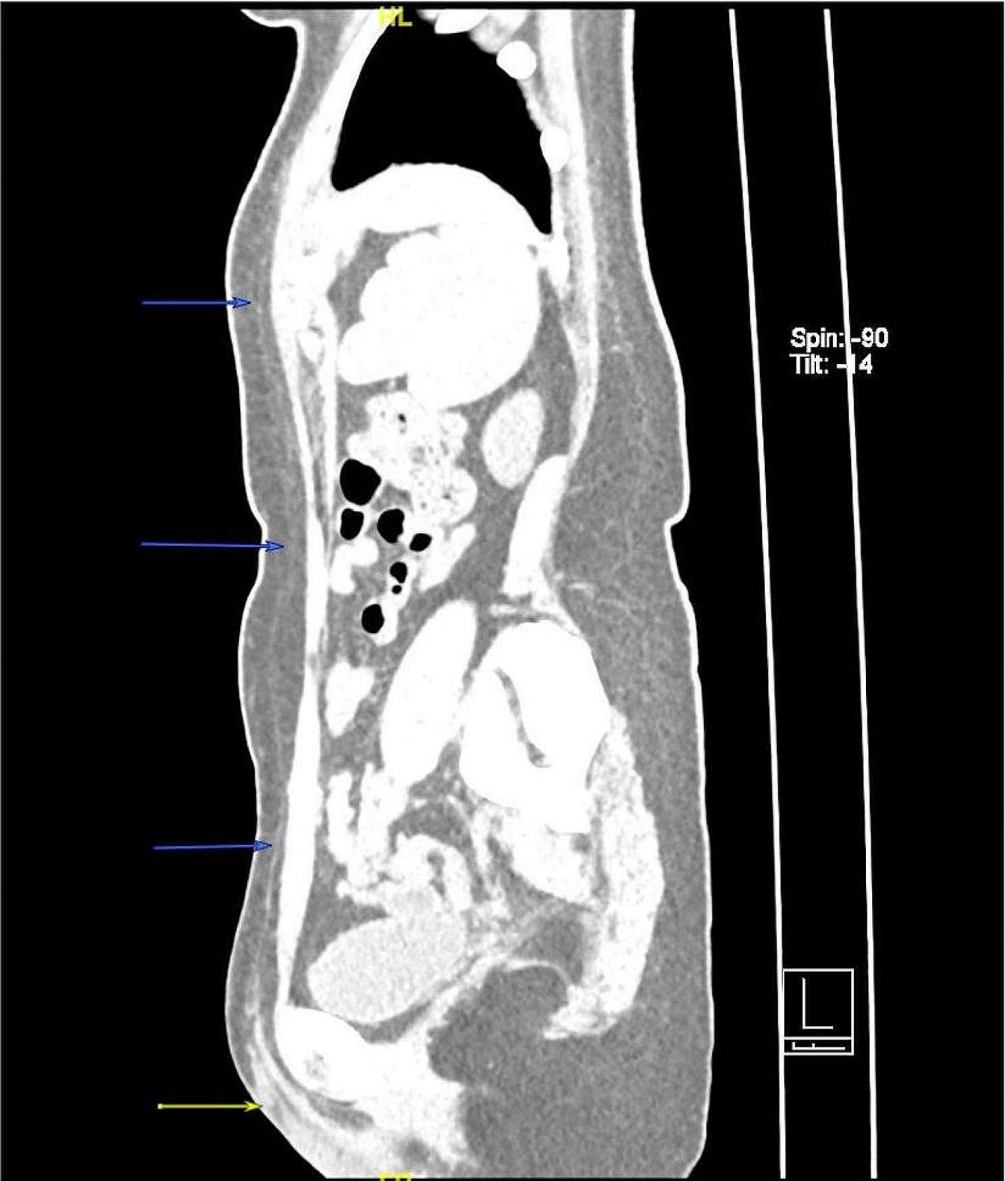
PAAG diffused within the subcutaneous fat; Blue arrow: Slightly high-density line-like shadows represents the subcutaneous sinus; Yellow arrow: a cystic area in the left vulva

**Fig. 3:**
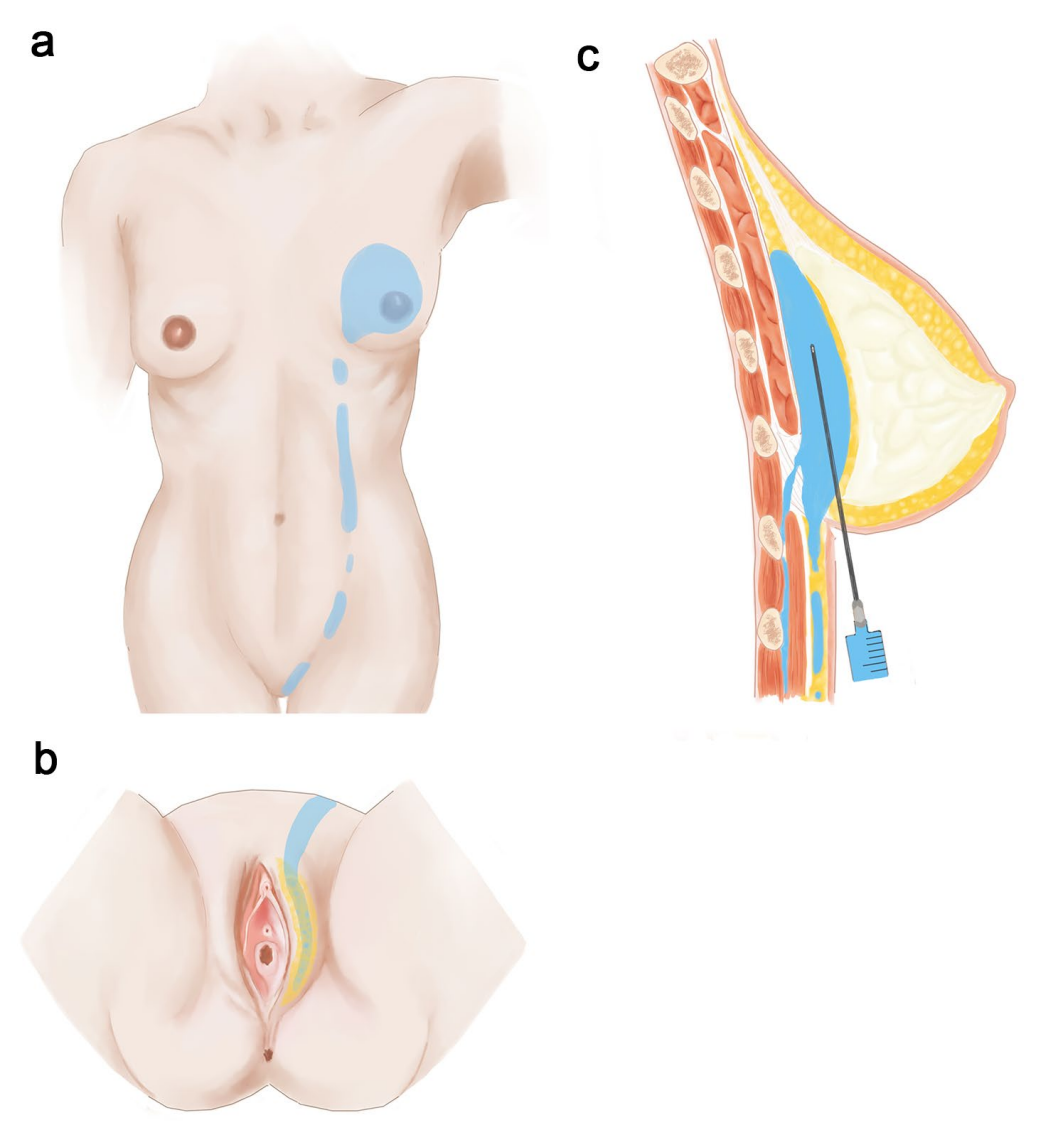
The possible route and mechanism of filler vulva migration; a.the possible PAAG migration route from breast to vulva; b.the pelvic migration of PAAG and the aggregation of it in vulva c.the process of PAAG injection and abdomen migration

On admission, there is a size of 10*3 cm abscess on her left labia majora with yellow pus draining from a fistula under the vulvar skin (Fig. 1 vulvar abscess). Ultrasound reveled a long cord-like hypoechoic change from the left breast to mons pubis. One part of the PAAG is located on the surface of the external oblique muscle and the other part is located below it. Slightly high-density shadows within subcutaneous fat below the left chest, abdomen and vulva were observed in computed tomography(CT) (Fig. 2 PAAG diffused within the subcutaneous fat). The patient presents with gradual reduction in size of the left breast, accompanied by the development of a vulvar abscess. Multiple imaging studies indicate migration of the left breast injection filler along the left chest and abdominal subcutaneous sinus tract to the left labia majora. There is a strong correlation between the “vulvar abscess” and the “breast injection material.” Upon further inquiry into the patient’s medical history, no evidence of diabetes, trauma, vulvar plastic surgery, or other relevant medical history was identified. Furthermore, no additional abnormalities were observed in imaging studies, effectively ruling out other pathological factors contributing to the abscess and subcutaneous sinus tract. We also drew a schematic diagram for this patient to demonstrate the possible route and mechanism of filler vulvar migration (Fig. 3 The possible route and mechanism of filler vulva migration).

**Fig. 4:**
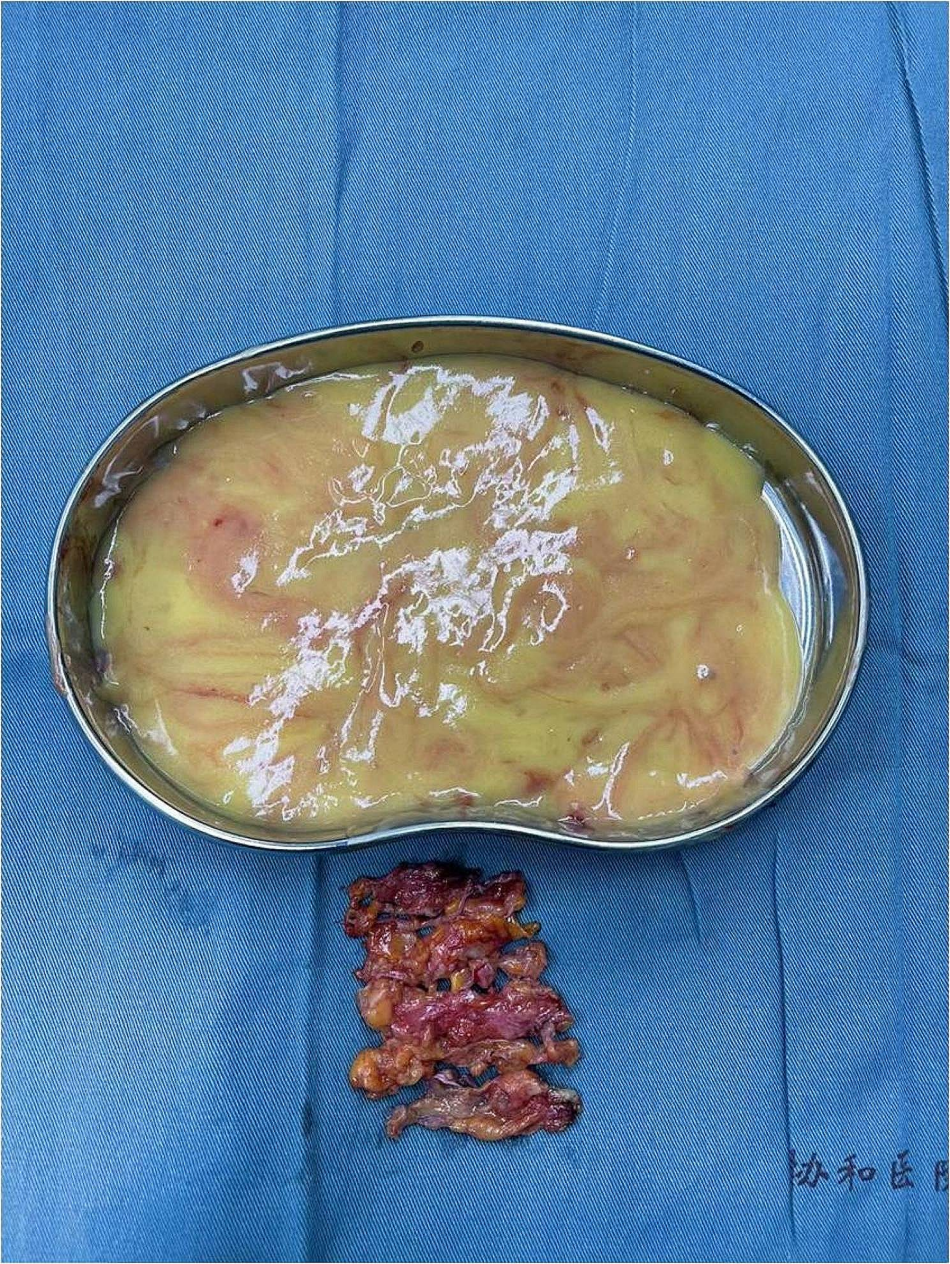
The removed yellow jelly gel, breast nodule and capsule during surgery

**Fig. 5:**
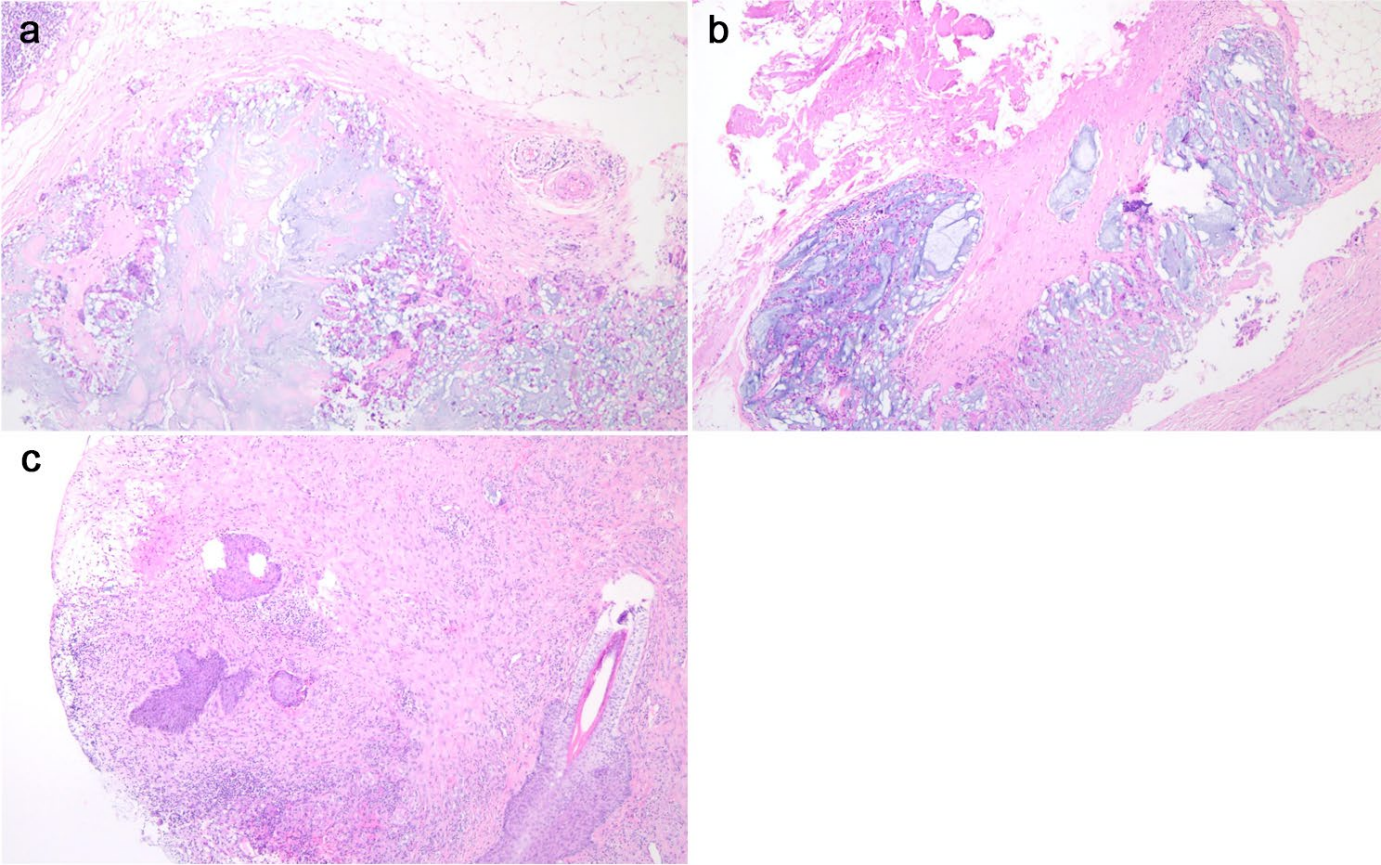
Histological features of left breast nodule, fibrous capsule, and vulvar sinus tissue; many tissue cells and foreign body giant cells in left breast nodule (a), fibrous capsule(b), and vulva sinus tissue (c)

**Fig. 6:**
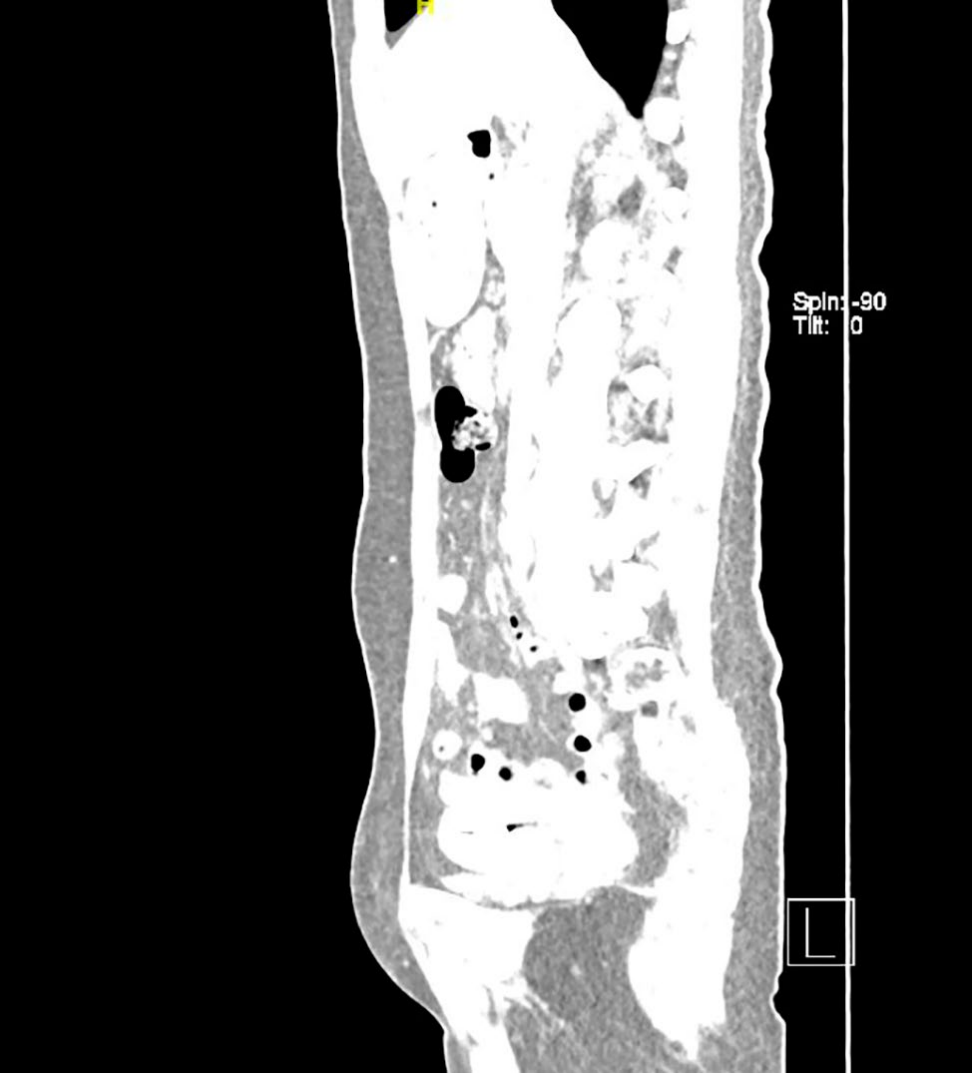
Postoperative thoraco-abdomin-pelvic CT showed no obvious residual filler

We soon scheduled a surgery for her. A 40 cm long sinus with gel was detected by intraoperative ultrasound, extending from the vulva to the left hypochondrial region. We used a semi-periareolar incision in left breast to explore the subcutaneous tissue and the gland. Then, three incisions were placed in the left labia majora (7 cm), suprapubic (5 cm), and left hypochondrial regions (5 cm) to scrutinize the areas where the filler was evidently present. The removed yellow jelly gel, breast nodule and fibrous capsule were shown in Fig. 4 (The removed yellow jelly gel, breast nodule and capsule during surgery). Vacuum sealing drainage (VSD) material was chosen to cover the wound, followed by continuous vacuum drainage. *E. coli*, *Staphylococcus epidermidis* and *Candida albicans* were isolated from vulva subcutaneous tissue specimens by bacteriological culture and the next generation sequencing (NGS). The VSD material was replaced a week later. When the wound showed fresh and rubicund surface granulation without sign of infection in the whole sinus, we removed the VSD materials, closed the lacuna and eliminated the polyacrylamide hydrogel of her right breast. The reports of postoperative pathology showed many tissue cells and foreign body giant cells in left breast nodule, fibrous capsule, and vulvar sinus tissue (Fig. 5 The histological features of left breast nodule, fibrous capsule, and vulvar sinus tissue). Postoperative thoraco-abdomin-pelvic CT scan showed that most of injected PAAG were cleaned up (Fig. 6 Postoperative thoraco-abdomin-pelvic CT). A month after hospitalization, this patient was discharged well without any symptoms. We also performed a telephone follow-up (there months later) and found that her symptoms of fever and pain did not recur.

## Discussion and conclusion

**Fig. 7:**
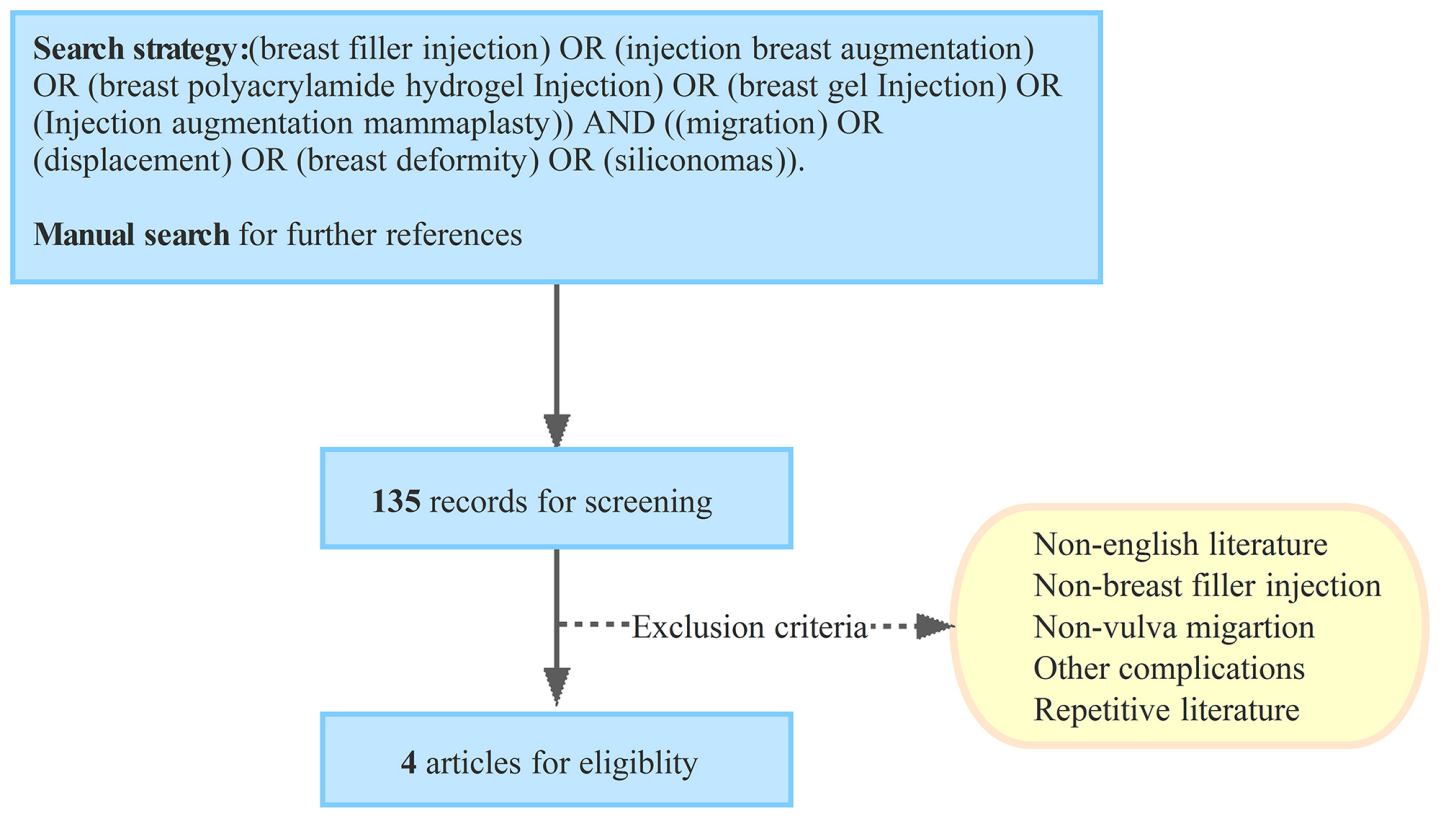
Flow diagram for study screening, selection and exclusion

A literature review was performed in October 2022 (Fig. 7 Flow diagram). The following search strategy were used in PubMed to obtain all the relevant English-language literature: (breast filler injection) or (injection breast augmentation) or (breast polyacrylamide hydrogel Injection) or (breast gel Injection) or (Injection augmentation mammaplasty)) and ((migration) or (displacement) or (breast deformity) or (siliconomas)).The relevant articles selected for this study included original articles and case reports/series that discussed the vulvar migration of breast filler injection. The articles excluded from this study were those with other different migration sites and those discussing other complications. The following data were extracted from the articles: year of publication, author(s), number of cases, medical history, filler substance and its migration and injection site, symptoms and signs, treatment, prognosis and key points (Supplement Table 1) .

There were only 4 cases of filler perineal area migration in total enrolled data according to our inclusion and exclusion criteria (Table 1) [4, 6, 8, 9]. All the four patients were female; the age range was from 26 to 49 years old, with an average of 36.5. Three of them received PAAG injection in breast, while one patient chose liquid silicone injection augmentation mammoplasty.

**Table 1:** The Data of the filler vulvar migration cases

Year	Author	Age	Filler	Radiologic feature			Interval time between filler injected and diagnosis	Clinical manifestations	Treatment	The duration between diagnosis and surgical intervention	Prognosis
				US	CT	MRI					
2005	Jeng et al. [9]	39	Liquid silicone	-	-	-	2 months	-	Vulvar mass removal surgery	1 months	Unknown
2017	Son et al. [6]	32	PAAG	Anechoic	Low-attenuated fluid-like material	High signal intensity on fat-saturated T1- and T2-weighted images	5 months	Fever, pain and hardness	Vulvar mass removal surgery and antibiotics therapy	4 months	Repeated pain and redness
2019	Zhang et al. [8]	49	PAAG	Hypoechoic	Low-attenuated fluid-like material	Cystic area in the left vulva	20 years	Swelling and tenderness of vulva mass	Vulvar mass removal surgery	Diagnosed during surgery	Repeated fever
2021	Nomoto et al. [4]	26	PAAG	-	-	-	4 years	Discomfort in the left ribs	Conservative treatment	-	Unknown

Migrated filler usually presents as a homogeneous hypoechoic or anechoic mass on sonography. CT scans displayed a low-density fluid-like signal. On fat-saturated T2-weighted images from MRI, it exhibits a high signal intensity. The time interval between onset of symptoms and surgery ranged from 2 months to 20 years. Onset of symptom of filler vulva migration were fever, pain, local oedema, tenderness and rib discomfort. Oral and/or i.v. antibiotic therapy did not prevent the worsening of the clinical condition. Three cases underwent vulvar mass removal surgery, but two of them still showed the symptoms of local redness, recurrent pain and recurrent fever after surgery. The time span from diagnosis to surgical intervention ranges from diagnosed during surgery to 4 months.

Although silicone gel or saline implants are preferred and reliable procedures, surgeons have tried to find a less invasive and simpler method for augmentation mammoplasty. Injection of PAAG offers a promising approach to soft tissue contour augmentation with only pinhole-like scars [10]. PAAG is reported to be a stable, nontoxic, nonallergenic, nonabsorbable and nonbiodegradable watery gel [11]. In 1997, PAAG produced by Ukraine was used to fill breast for the first time in the China [12]. PAAG injections later have been shown to be potentially dangerous, causing a large number of women suffering from postoperative complications [13–17]. Although it was later prohibited from production and clinical application, more than 200,000 women in China have been underwent breast augmentation by PAAG injection during the last ten years [18].

Implant migration is one of the unacceptable complications to patients. However, according to a multicenter study, PAAG migration was found in every third patient who is associated with larger gel volumes and longer injection times [13]. Ultrasound can precisely identify the location of the injected PAAG, which appears as a predominantly anechoic and hypoechoic collection; On CT scan, filler appears as fluid attenuation accompanied by thin rim enhancement; PAAG on MRI sequences present as hypointense on T1-weighted sequences and hyperintense on T2-weighted sequences [6, 19, 20]. The radiologic features in our own case are consistent with the reported in literatures and confirmed through surgery.

The reasons of vulvar filler migration are unclear. A previous experimental assessment showed the formation of capsule material around the PAAG, which was integrated into the tissue [10]. However, the hydrophilicity and fine granularity of PAAG does not seem to produce a definite thick fibrous capsule for long-lasting breast augmentation effect [12, 21, 22]. Furthermore, PAAG fillers can induce a high inflammatory response before surrounded by a capsule, decomposing the normal tissue structures and resulting in distant migration [4, 23].

The inframammary crease is the most common injection site, which argued by some to be the anatomical location of the inframammary fold ligament [24]. When a large volume of PAAG or liquid silicon were injected in retromammary space by a large-bore cannula, it is assumed that the filler could exceed the compact inframammary fold through the puncture tunnel and spread to other parts with loose connective tissues. Consequently, the fat layers of these regions would be invaded and a big sinus would be therefore formed after the necrotic fat absorbed [25]. In addition, inframammary fold ligament originates from the confluence and thickening of the two converging layers of the superficial fascia and composed mainly of collagen and elastic fibers [24]. Once fillers are injected in different layers of the inframammary fold ligament, the pressure produced by muscle contraction and the large volume of injected filler will promote the PAAG or liquid silicon to more easily migrate to a space with less resistance, such as abdominal and vulvar wall (Fig. 3).

The PAAG gel is composed of approximately 2.5% cross-linked polyacrylamide and non-pyrogenic water [26]. Even when exposed to enzymes, bacteria, and oxidizing agents, the gel demonstrates remarkable stability [26]. It also has the ability to absorb bodily fluids and exudates, creating a nutrient-rich environment conducive to bacterial growth [27]. When PAAG becomes a good medium for bacterial growth mixing with pathogens, it could flow along the submammary space, resulting in the secondary infection of surrounding tissues [27]. Once the site suffers from the chronic infection, sinus tract would easily form in the skin and hard to heal, sometimes leading to life-threatening septic shock or sepsis [18, 28]. The vulvar abscess could be the result of direct bacterial extension through the sinus tract. Early infection may be related to lax operation in the aseptic technique, Breast duct injury and allergic reactions. However, the infection also occurred many years later in the literature review. *E. coli*, *Staphylococcus epidermidis* and *Candida* were found in our own report. All of them are opportunistic bacterial pathogens. Decreased immune response and opportunistic pathogens entry the exposed wound following vulvar mass resection may have been the reason of late repeated infections of this case [20, 29]. Surgical drainage and intravenous antibiotics are recommended for management [30].

Migration is also a known side effect for liquid silicone breast injections. it is thought that silicone can migrate through hematogenous or lymphatic routes [31]. Various distant migration sites of silicone from breast are reported, including the upper extremity, groin and thoracic cavity [32, 33]. Lymphatic migration of silicone in the groin lymph node is considered as a potential route for dissemination. The transit of PAAG from breast tissue to lymph nodes via lymphatic channels may also be an important factor in the metastatic process. Moreover, factors such as trauma, gravity and external pressure were believed to play essential parts in the migration of PAAG from the chest wall [4, 8].

It is reported that vulvar mass removal surgery in previous patients presents unsatisfactory results [6, 8, 9]. A surgical debridement operation permits a removal of gel as much thoroughly as possible. However, there are few methods for thoroughly treating the huge cavity caused by PAAG migration. A primary treatment recommendation based on our experience: for patients with a distant migration of filler, several incisions should be made at sites where the presence of the filler is evident on intraoperative ultrasound to remove the maximum amount of filler, reducing the damage of the toxic substances to the human and achieving a rapid tissue healing. The surrounding capsule and degenerated tissues should be completely removed and pathological examination should be conducted. It is necessary to close the sinus to promote healing, continuous postoperative vacuum sealing drainage (VSD) and irrigation can be applied. For patients with suspected infection, bacterial cultures, NGS if necessary, should be arranged, and antibiotics should be used based on the drug sensitivity tests results after surgery.

PAAG gel migration is a global complex problem and requires individualized treatment. Here we provide an analysis of the reasons of PAAG vulva migration, hoping to improve the comprehensive understanding of this complications. Our case and literature review illustrate the need for a thorough physical examination for patients with a history of gel breast injection when vulva filler migration is suspected.

### Electronic supplementary material

Below is the link to the electronic supplementary material.

Supplementary Material 1

## Data Availability

All data generated or analyzed during this study are included in this published article.

## Declarations

### Human or animal participants

This article does not contain any studies with human participants or animals performed by any of the authors.

## Abbreviations

CFDA: China Food and Drug Administration
CT: Computed Tomography
NGS: Next generation sequencing
PAAG: Polyacrylamide hydrogel
VSD: Vacuum sealing drainage
