# Supplementary material for: Vulvar migration of injected polyacrylamide hydrogel following breast augmentation: a case report and literature review
Source: BMC Womens Health. 2024 Mar 2;24:152. doi: 10.1186/s12905-024-02998-0 (PMC10909281; doi:10.1186/s12905-024-02998-0)
Supplement: Supplementary file 1 — Supplementary Material 1 [file 12905_2024_2998_MOESM1_ESM.docx]

Supplementary table 1. The Data of the Enrolled Cases

| **Year** | **Author** | **No of cases** | **History** | **Filler** | **Migration Site** | **Injection Site** | **Other complications** | **Treatment** | **Prognosis** | **Key point** |
| --- | --- | --- | --- | --- | --- | --- | --- | --- | --- | --- |
| 1978 | Capozzi et al^1^ | 1 | - | Ruptured silicone  implant | Subcutaneous plane | - | - | Removal surgery | Unknown | - |
| 1978 | Celli et al^2^ | 1 | - | Liquid silicone | Lung | Unknown | Dyspnea | Conservative treatment | Symptoms relived | - |
| 1981 | Mason et al.^3^ | 1 | - | Ruptured silicone  implant | Left upper arm | - | - | Biopsy and removal surgery | Unknown | - |
| 1983 | Chastre et al.^4^ | 2 | Transsexual | Liquid silicone | Lung | Unknown | Fever, cough, bloody sputum, and shortness of breath | Conservative treatment | Symptoms relived |  |
| 1983 | Foster et al.^5^ | 2 | - | Ruptured silicone  implant | Upper arm | - | - | Biopsy and removal surgery | Satisfaction |  |
| 1988 | Truong et al.^6^ | 5 |  | Liquid silicone and ruptured silicone  implant(N=1) | Axilla; supraclavicular | Unknown | - | Biopsy and removal surgery | Unknown |  |
| 1992 | Okub et al.^7^ | 2 | - | Paraffin(N=1)；  liquid silicone(N=1) | Axilla; inguinal; | Unknown | Ulceration of the lower abdomen; Breast cancer | Removal surgery(N=1)；Biopsy(N=1) | Undergoing chemotherapy two years after the operation; Died half a year after the operation(N=1) | - |
| 1993 | Chen et al.^8^ | 3 | - | Liquid silicone | Lung,liver,brain,kidney | Unknown | Hemoptysis, dyspnea, nausea, vomiting, fever exertional dyspnea | Conservative treatment | Died (N=1) |  |
| 1994 | Matsuba et al.^9^ | 1 | - | Liquid silicone | Lung | Unknown | Dyspnea, dry cough, and bilateral pleuritic chest  pain | Conservative treatment | Symptoms relived |  |
| 1994 | Lai et al.^10^ | 7 | - | Liquid silicone | Lung | Unknown | Hemoptysis, dyspnea | Conservative treatment | Symptoms relived |  |
| 1996 | Ooi et al.^11^ | 1 | - | Paraffin | Thoracic wall, Mediastinum | Unknown | Bloodstained sputum；exertional dyspnea | Breast removal surgery and conservative treatment | Satisfaction and symptoms relived | - |
| 1998 | Meyer et al.^12^ | 1 | Diabetes; Breast Cancer | Ruptured silicone  implant | Eyelid | - | Stifiness in the joints and hands | Biopsy of the eyelids | - | - |
| 2001 | Ho et al.^13^ | 1 | - | Paraffin | Chest wall | Unknown | Pain | Removal surgery | Unknown |  |
| 2005 | Jeng et al.^14^ | 1 | - | Liquid silicone | Vulvar | Unknown | - | Vulvar mass removal surgery | Unknown | - |
| 2005 | Schmid et al.^15^ | 1 | HIV | Liquid silicone | Lung | Unknown | Cough, progressive severe  dyspnea, chest tightness, and fever | Conservative treatment | Symptoms relived |  |
| 2006 | Cheng et al.^16^ | 8 | Unknown | PAAG | The periphery of the mammary glands or epigastrium | Unknown | Unknown | Removal surgery | Unknown | - |
| 2006 | Markopoulos et al.^17^ | 1 | - | Paraffin | Abdominal skin | Unknown | Unknown | Bilateral mastectomy; Immediate reconstruction failed; Late reconstruction in 10 months | Poor cosmetic result |  |
| 2008 | Lui et al.^18^ | 5 | Unknown | PAAG | Intrathoracic extension((N=2)  Abdominal wall(N=3) | Inframammary crease (the most common site) | Unknown | Unknown | Unknown | Intrathoracic extension of PAAG was first reported. |
| 2009 | Sagi et al.^19^ | 1 | - | Ruptured silicone  implant | shins and ankles | - | Unknown | Biopsy | - |  |
| 2010 | McCleave et al.^20^ | 2 | - | HA | Axilla; submammary | Unknown | - | Removal surgery | Unknown |  |
| 2010 | Vila et al.^21^ | 1 | - | Ruptured silicone  implant | Lung | - | - | Removal surgery | Unknown |  |
| 2011 | Unukovych et al.^22^ | 39 | Unknown | PAAG | Submammary (N=12) Axilla (N=10) Sternum(N=3) Abdominal wall(N=1) Two or more zones(N=13) | Unknown | Unknown | Removal surgery | Satisfaction of the majority of patients | - |
| 2011 | Khedher et al.^23^ | 2 | Unknown | PAAG | Chest wall; infraclavicular space | Inframammary fold | Unknown | Conservative treatment | Unknown | - |
| 2011 | Luo et al.^24^ | 21 | Unknown | PAAG | Axilla, abdomen, pubes. | Unknown | Unknown | Removal surgery | Satisfaction of the majority of patients | - |
| 2012 | Do.et al.^25^ | 1 | - | PAAG | The center of the chest | - | - | Aspiration | Unknown | - |
| 2012 | Lee et al.^26^ | 1 | - | Prolamine | Upper abdomen | Axilla | - | Removal surgery | Satisfaction | - |
| 2013 | Chuangsuwanich et al.^27^ | 1 | - | Liquid silicone | Axillary | Unknown | Pain | Removal surgery | Breast reconstruction 3 mouths later. | - |
| 2014 | Ishii et al.^28^ | 3 | - | HA | Inframammary fold and precordium | Unknown | - | Aspiration | Reoccurrence in two patients |  |
| 2014 | Wu et al.^29^ | 1 | - | PAAG | Chest and abdomen | Unknown | Swelling and pain | Removal surgery and VSD | Satisfaction | - |
| 2014 | Wong et al.^30^ | 1 | - | Paraffin | Right upper quadrant | Unknown | - | Biopsy | - | - |
| 2015 | Chen et al.^31^ | 102 | Unknown | PAAG | Axilla, thoracic and abdomen wall | Unknown | Unknown | Removal surgery and VSD | Satisfaction of the majority of patients | - |
| 2015 | Hilton et al.^32^ | 1 | - | Liquid silicone | Left supraclavicular; left chest wall; along the proximal; medial part of the left arm | Unknown | Septic; left biceps region and left upper limb cellulitis; staphylococcus bacteraemia | Removal surgery and reconstruction | Unknown | - |
| 2015 | Tanaka et al.^33^ | 1 | - | Ruptured silicone  implant | pleural | - | Fever; chest and back pain | Removal surgery | Satisfaction | Intrapulmonary or intrathoracic silicone migration |
| 2015 | Grella et al.^34^ | 1 | - | Methacrylate | Sternal region | Inframammary fold | Chest pain | Removal surgery | Satisfaction | The first case reporting the use of methacrylate for large volume injections to the breast |
| 2016 | Oh et al.^35^ | 1 | - | Ruptured silicone  implant | Inguinal and knee areas | - | Bulging and pain | Removal surgery | Symptoms relived | - |
| 2016 | Chen et al.^36^ | 50 | Unknown | PAAG | Unknown | Unknown | Unknown | Removal surgery | Satisfaction of the majority of patients | = |
| 2017 | Son et al.^37^ | 3 | - | PAAG | Left upper parasternal area; axillae, the subclavian triangles and the intercostal space of the left parasternal area; Vulva | Unknown | Fever, pain and hardness | Removal surgery | Unknown | - |
| 2018 | Chen et al.^38^ | 1 | Breast cancer | Ruptured silicone  implant | Eyelids, orbit, face, trunk, arms, and legs |  | Ptosis, proptosis, and vision loss;  muscle and joint pains, stiffness, and fatigue | Orbital and periorbital debulking procedure; breast implant removal | Dramatic improvement of her periorbital and orbital disease, as well as significant regression of her forearm and leg granulomas. | the first case of disseminated silicone granulomatosis with robust orbito facial involvement due to rupture of breast implants |
| 2019 | Zhang et al.^39^ | 1 | - | PAAG | Vulva | Unknown | Swelling and tenderness | The resection of the vulvar mass and VSD | Swelling of the bilateral chest wall with fever |  |
| 2019 | Hudacko et al.^40^ | 2 | Iron deficiency anemia secondary to menometrorrhagia(N=1）；  cholecystectomy and bipolar disorder(N=1） | Ruptured silicone  implant | Liver | - | Malaise and dyspnea on exertion | Liver biopsy | No further treatment |  |
| 2020 | Namgoong et al.^41^ | 12(including migration following hip gel injection) | Unknown | PAAG | Unknown | Unknown | Unknown | Removal surgery | Satisfaction of the majority of patients | - |
| 2020 | Qian et al^42^ | 20 | Unknown | PAAG | Unknown | Unknown | Unknown | Removal surgery | Satisfaction of the majority of patients |  |
| 2020 | Yang et al.^43^ | 19 | Unknown | PAAG | Infraclavicula, hypochondria, abdominal wall, anterior sternum, axilla, and even the posterior chest wall | Unknown | Unknown | Unknown | Unknown | - |
| 2020 | Kim et al.^44^ | 1 | - | PAAG | Axillae | Unknown | Pain and tenderness in both breast | Removal surgery | Breast reconstruction 3 mouths later. | - |
| 2020 | Zhang et al.^45^ | 135 | Unknown | PAAG | Unknown | Unknown | Unknown | Removal surgery | Satisfaction | - |
| 2020 | He et al.^46^ | 78(116 breasts) | Unknown | PAAG | Axilla and upper arm(N=23)  Lower back(N=27)  Abdomen or pubis(N=36)  Contralateral breast(N=11)  Two or more directions(N=9) | Unknown | Unknown | Removal surgery | Satisfaction（9mouths） | In breasts with superior and/or lateral displacements, the gel material was found in both the retroglandular and submuscular spaces. In contrast, no material was found in the submuscular layer in other types. |
| 2020 | Trignano et al.^47^ | 1 | - | HA | Axillary | Mostly in the upper poles in the right breast and the medial and inferior poles in the left one. | Pain and swelling in the right axilla | Removal surgery | Pain relived | Hyaluronic acid accumulation in lymph nodes. |
| 2021 | Maw et al.^48^ | 1 | Unknown | PAAG | Interpectoral region | Unknown | Unknown | Unknown | Unknown |  |
| 2021 | Chalcarz et al^49^ | 1 | - | PAAG | below the inframammary fold | Unknown | - | Removal surgery | Unknown | - |
| 2021 | Wang et al.^50^ | 1 | Familial vitiligo | PAAG | Infraclavicular, right thoracic wall, perineum, and left side of the upper back, nearly the entire anterior abdomen | Unknown | - | Conservative treatment | - | - |
| 2021 | Munagala et al.^51^ | 1 | HIV | Liquid silicone | Midline anterior chest wall | Unknown | Worsening shortness of breath and chest heaviness | Removal surgery | No short of breath or heavy | This patient was initially diagnosed with HIV lipodystrophy. |
| 2021 | Nomoto et al.^52^ | 4 | - | Copolyamide fillers | Back(N=1);  Vulva(N=1); under the pectoralis major muscle(N=1);  lower abdomen(N=1); | Unknown | Discomfort in the left ribs(N=1) | Removal surgery (N=1);  Needle aspiration and suction; (N=1)  conservative treatment; (N=1) | Unknown | Filler was demonstrated retention in the axilla |
| 2022 | Cordero rt al^53^ | 2 | Unknown | Liquid silicone(N=1); Unknown N=1) | Midline anterior chest wall | Unknown | Unknown | Removal surgery and staged reconstruction | Delayed wound healing | Transgender Women |
| 2022 | Neerukonda et al.^54^ | 2 | - | Ruptured silicone  implant | Eyelid crease | - | Invasive ductal carcinoma;  Mechanical ectropion of all eyelids(N=1) | Removal surgery | Unknown | Silicone can migrate through both soft tissue planes or hematogenous or lymphatic routes |
| 2022 | Gao et al.^55^ | 16 | Unknown | PAAG | Unknown | Unknown | Unknown | Removal surgery | Unknown | - |
| 2022 | Choi et al.^56^ | 1 | - | PAAG | left axilla, chest wall, abdominal wall, and peritoneum | Unknown | - | US–guided aspiration | Unknown | - |

Reference

1. Capozzi A, Du Bou R, Pennisi VR. Distant migration of silicone gel from a ruptured breast implant. Case report. Plast Reconstr Surg 1978; 62 (2): 302-303. doi: 10.1097/00006534-197808000-00038.

2. Celli B, Textor S, Kovnat DM. Adult respiratory distress syndrome following mammary augmentation. Am J Med Sci 1978; 275 (1): 81-85. doi: 10.1097/00000441-197801000-00009.

3. Mason J, Apisarnthanarax P. Migratory silicone granuloma. Arch Dermatol 1981; 117 (6): 366-367.

4. Chastre J, Basset F, Viau F, Dournovo P, Bouchama A, Akesbi A, et al. Acute pneumonitis after subcutaneous injections of silicone in transsexual men. N Engl J Med 1983; 308 (13): 764-767. doi: 10.1056/nejm198303313081307.

5. Foster WC, Springfield DS, Brown KL. Pseudotumor of the arm associated with rupture of silicone-gel breast prostheses. Report of two cases. J Bone Joint Surg Am 1983; 65 (4): 548-551.

6. Truong LD, Cartwright J, Jr., Goodman MD, Woznicki D. Silicone lymphadenopathy associated with augmentation mammaplasty. Morphologic features of nine cases. Am J Surg Pathol 1988; 12 (6): 484-491. doi: 10.1097/00000478-198806000-00009.

7. Okubo M, Hyakusoku H, Kanno K, Fumiiri M. Complications after injection mammaplasty. Aesthetic Plast Surg 1992; 16 (2): 181-187. doi: 10.1007/bf00450611.

8. Chen YM, Lu CC, Perng RP. Silicone fluid-induced pulmonary embolism. Am Rev Respir Dis 1993; 147 (5): 1299-1302. doi: 10.1164/ajrccm/147.5.1299.

9. Matsuba T, Sujiura T, Irei M, Kyan Y, Kunishima N, Uchima H, et al. Acute pneumonitis presumed to be silicone embolism. Intern Med 1994; 33 (8): 481-483. doi: 10.2169/internalmedicine.33.481.

10. Lai YF, Chao TY, Wong SL. Acute pneumonitis after subcutaneous injections of silicone for augmentation mammaplasty. Chest 1994; 106 (4): 1152-1155. doi: 10.1378/chest.106.4.1152.

11. Ooi GC, Peh WC, Ip M. Migration and lymphatic spread of calcified paraffinomas after breast augmentation. Australas Radiol 1996; 40 (4): 404-407. doi: 10.1111/j.1440-1673.1996.tb00435.x.

12. Meyer DR, Bui HX, Carlson JA, Ratliff CD, Guevarra MC, DelRosario AD, et al. Silicon granulomas and dermatomyositis-like changes associated with chronic eyelid edema after silicone breast implant. Ophthalmic Plast Reconstr Surg 1998; 14 (3): 182-188. doi: 10.1097/00002341-199805000-00007.

13. Ho WS, Chan AC, Law BK. Management of paraffinoma of the breast: 10 years' experience. Br J Plast Surg 2001; 54 (3): 232-234. doi: 10.1054/bjps.2000.3533.

14. Jeng CJ, Ko ML, Wang TH, Huang SH. Vulvar siliconoma migrating from injected silicone breast augmentation. Bjog 2005; 112 (12): 1659-1660. doi: 10.1111/j.1471-0528.2005.00761.x.

15. Schmid A, Tzur A, Leshko L, Krieger BP. Silicone embolism syndrome: a case report, review of the literature, and comparison with fat embolism syndrome. Chest 2005; 127 (6): 2276-2281. doi: 10.1378/chest.127.6.2276.

16. Cheng NX, Xu SL, Deng H, Ding XB, Zhang XM, Wu DH, et al. Migration of implants: a problem with injectable polyacrylamide gel in aesthetic plastic surgery. Aesthetic Plast Surg 2006; 30 (2): 215-225. doi: 10.1007/s00266-005-0081-5.

17. Markopoulos C, Mantas D, Kouskos E, Antonopoulou Z, Revenas C, Yiacoumettis A. Paraffinomas of the breast or oleogranulomatous mastitis-a rare entity. Breast 2006; 15 (4): 540-543. doi: 10.1016/j.breast.2005.08.036.

18. Lui CY, Ho CM, Iu PP, Cheung WY, Lam HS, Cheng MS, et al. Evaluation of MRI findings after polyacrylamide gel injection for breast augmentation. AJR Am J Roentgenol 2008; 191 (3): 677-688. doi: 10.2214/ajr.07.2733.

19. Sagi L, Baum S, Lyakhovitsky A, Barzilai A, Shpiro D, Trau H, et al. Silicone breast implant rupture presenting as bilateral leg nodules. Clin Exp Dermatol 2009; 34 (5): e99-101. doi: 10.1111/j.1365-2230.2008.03196.x.

20. McCleave MJ, Grover R, Jones BM. Breast enhancement using Macrolane™: a report of complications in three patients and a review of this new product. J Plast Reconstr Aesthet Surg 2010; 63 (12): 2108-2111. doi: 10.1016/j.bjps.2010.02.021.

21. Paredes Vila S, Gonzalez Barcala FJ, Suarez Antelo J, Moldes Rodriguez M, Abdulkader Nallib I, Valdes Cuadrado L. Pneumonitis caused by silicone gel following breast implant rupture. Ir J Med Sci 2010; 179 (1): 141-145. doi: 10.1007/s11845-009-0369-6.

22. Unukovych D, Khrapach V, Wickman M, Liljegren A, Mishalov V, Patlazhan G, et al. Polyacrylamide gel injections for breast augmentation: management of complications in 106 patients, a multicenter study. World J Surg 2012; 36 (4): 695-701. doi: 10.1007/s00268-011-1273-6.

23. Khedher NB, David J, Trop I, Drouin S, Peloquin L, Lalonde L. Imaging findings of breast augmentation with injected hydrophilic polyacrylamide gel: patient reports and literature review. Eur J Radiol 2011; 78 (1): 104-111. doi: 10.1016/j.ejrad.2009.09.021.

24. Luo SK, Chen GP, Sun ZS, Cheng NX. Our strategy in complication management of augmentation mammaplasty with polyacrylamide hydrogel injection in 235 patients. J Plast Reconstr Aesthet Surg 2011; 64 (6): 731-737. doi: 10.1016/j.bjps.2010.10.004.

25. Do ER, Shim JS. Long-term Complications from Breast Augmentation by Injected Polyacrylamide Hydrogel. Arch Plast Surg 2012; 39 (3): 267-269. doi: 10.5999/aps.2012.39.3.267.

26. Lee SI, Han JK, Hwang K, Park S, Jang SA. Analysis of migrated hydrogel used for breast augmentation revealed prolamin (a cereal seed storage protein). Aesthetic Plast Surg 2012; 36 (1): 207-212. doi: 10.1007/s00266-011-9770-4.

27. Chuangsuwanich A, Warnnissorn M, Lohsiriwat V. Siliconoma of the breasts. Gland Surg 2013; 2 (1): 46-49. doi: 10.3978/j.issn.2227-684X.2013.02.05.

28. Ishii H, Sakata K. Complications and management of breast enhancement using hyaluronic acid. Plast Surg (Oakv) 2014; 22 (3): 171-174.

29. Wu J, Zhang X, Zhao Q, Mao D, Lu X. Vacuum sealing drainage in the treatment of migrated polyacrylamide hydrogel after breast augmentation: a case report. Breast Care (Basel) 2014; 9 (4): 273-275. doi: 10.1159/000365954.

30. Wong KT, Lee PS, Chan YL, Chow LT. Paraffinoma in anterior abdominal wall mimicking liposarcoma. Br J Radiol 2003; 76 (904): 264-267. doi: 10.1259/bjr/31110098.

31. Chen L, Sha L, Huang SP, Li SR, Wang ZX. Treatment for displacement of PAAG mixture after injection augmentation mammoplasty. Int J Clin Exp Med 2015; 8 (3): 3360-3370.

32. Hilton JD, Steinke K. Extensive migration of injected free liquid silicone for breast augmentation with related major complications. BJR Case Rep 2015; 1 (2): 20150098. doi: 10.1259/bjrcr.20150098.

33. Tanaka T, Tao H, Hayashi T, Yoshiyama K, Furukawa M, Yoshida K, et al. Disseminated Pleural Siliconoma Mimicking Malignant Pleural Mesothelioma. Ann Thorac Surg 2015; 100 (6): 2339-2340. doi: 10.1016/j.athoracsur.2015.03.043.

34. Grella R, Almadori A, D'Ari A, Romanucci V, D'Andrea F. Management of complication after breast augmentation with methacrylate. Int J Surg Case Rep 2015; 15 17-20. doi: 10.1016/j.ijscr.2015.06.038.

35. Oh JH, Song SY, Lew DH, Lee DW. Distant Migration of Multiple Siliconomas in Lower Extremities following Breast Implant Rupture: Case Report. Plast Reconstr Surg Glob Open 2016; 4 (10): e1011. doi: 10.1097/gox.0000000000001011.

36. Chen B, Song H. Management of Breast Deformity After Removal of Injectable Polyacrylamide Hydrogel: Retrospective Study of 200 Cases for 7 Years. Aesthetic Plast Surg 2016; 40 (4): 482-491. doi: 10.1007/s00266-016-0646-5.

37. Son MJ, Ko KH, Jung HK, Koh JE, Park AY. Complications and Radiologic Features of Breast Augmentation via Injection of Aquafilling Gel. J Ultrasound Med 2018; 37 (7): 1835-1839. doi: 10.1002/jum.14527.

38. Chen TA, Mercado CL, Topping KL, Erickson BP, Cockerham KP, Kossler AL. Disseminated silicone granulomatosis in the face and orbit. Am J Ophthalmol Case Rep 2018; 10 32-34. doi: 10.1016/j.ajoc.2018.01.037.

39. Zhang MX, Li SY, Xu LL, Zhao BW, Cai XY, Wang GL. Repeated lumps and infections: A case report on breast augmentation complications. World J Clin Cases 2019; 7 (20): 3322-3328. doi: 10.12998/wjcc.v7.i20.3322.

40. Hudacko R, Anand K, Gordon R, John T, Catalano C, Zaldana F, et al. Hepatic Silicone Granulomas Secondary to Ruptured Breast Implants: A Report of Two Cases. Case Reports Hepatol 2019; 2019 7348168. doi: 10.1155/2019/7348168.

41. Namgoong S, Kim HK, Hwang Y, Shin SH, You HJ, Kim DW, et al. Clinical Experience with Treatment of Aquafilling Filler-Associated Complications: A Retrospective Study of 146 Cases. Aesthetic Plast Surg 2020; 44 (6): 1997-2007. doi: 10.1007/s00266-020-01889-7.

42. Qian B, Xiong L, Guo K, Wang R, Yang J, Wang Z, et al. Comprehensive management of breast augmentation with polyacrylamide hydrogel injection based on 15 years of experience: a report on 325 cases. Ann Transl Med 2020; 8 (7): 475. doi: 10.21037/atm.2020.03.68.

43. Yang Y, Li S, He J, Zhao X, Chen W, Dai X, et al. Clinicopathological Analysis of 90 Cases of Polyacrylamide Hydrogel Injection for Breast Augmentation Including 2 Cases Followed by Breast Cancer. Breast Care (Basel) 2020; 15 (1): 38-43. doi: 10.1159/000499832.

44. Kim HJ, Lee SJ, Lee JH, Shin SH, Kim SH, Kim JH, et al. Breast reconstruction after complications following breast augmentation with massive filler injections. Medicine (Baltimore) 2020; 99 (33): e21516. doi: 10.1097/md.0000000000021516.

45. Zhang F, Wang X, Guo H. Different Types of Breast Deformity Induced by Two Types of Polyacrylamide Hydrogel and Corresponding Treatment. Aesthetic Plast Surg 2020; 44 (3): 726-734. doi: 10.1007/s00266-020-01626-0.

46. He J, Wang T, Dong J. Classification and Management of Polyacrylamide Gel Migration After Injection Augmentation Mammaplasty: A Preliminary Report. Aesthetic Plast Surg 2020; 44 (5): 1516-1521. doi: 10.1007/s00266-020-01912-x.

47. Trignano E, Baccari M, Pili N, Serra PL, Rubino C. Complications after breast augmentation with hyaluronic acid: a case report. Gland Surg 2020; 9 (6): 2193-2197. doi: 10.21037/gs-20-448.

48. Maw AM, Sood S, Schembri GP. Case Report of 18F-FDG PET/CT Features of Polyacrylamide Hydrogel Mammoplasty. Clin Nucl Med 2021; 46 (4): e206-e207. doi: 10.1097/rlu.0000000000003403.

49. Chalcarz M, Żurawski J. Injection of Aquafilling(®) for Breast Augmentation Causes Inflammatory Responses Independent of Visible Symptoms. Aesthetic Plast Surg 2021; 45 (2): 481-490. doi: 10.1007/s00266-020-01949-y.

50. Wang B, Sun J, Tong J. Breast contracture and skin sclerosis following 20 years of polyacrylamide hydrogel migration in a patient with familial vitiligo: a case report. BMC Surg 2021; 21 (1): 104. doi: 10.1186/s12893-021-01097-3.

51. Munagala R, Mishra P, Chakravartty A, Bhatt AN, Keshavamurthy J. Diagnosis and Management of Rare Siliconomas in an HIV Patient. Cureus 2021; 13 (4): e14645. doi: 10.7759/cureus.14645.

52. Nomoto S, Hirakawa K, Ogawa R. Safety of Copolyamide Filler Injection for Breast Augmentation. Plast Reconstr Surg Glob Open 2021; 9 (2): e3296. doi: 10.1097/gox.0000000000003296.

53. Cordero DM, Srinivasa D, Barnes LL, Ray E, Terry MJ. Surgical Treatment of Granulomatous Breast Deformities Caused by Injection of Foreign Substances in Transgender Women: A Case Series and Algorithm. Plast Reconstr Surg 2022; 149 (6): 1312-1316. doi: 10.1097/prs.0000000000009092.

54. Neerukonda VK, Lefebvre D, Chatson GP, Stagner AM. Silicone Granulomas of the Eyelids-A Case Series Illustrating a Distant Migratory Phenomenon. Ophthalmic Plast Reconstr Surg 2022; doi: 10.1097/iop.0000000000002255.

55. Gao Q, Zhai P, Qi J, Yang Z, Hu Y, Yuan X, et al. Breast Augmentation with Autologous Fat Grafting Immediately after Removal of Polyacrylamide Hydrogel and Fibrotic Capsule in 162 Patients. Breast Care (Basel) 2022; 17 (4): 377-384. doi: 10.1159/000522616.

56. Choi YJ, Lee IS, Song YS, Choi KU, Ahn HY. Distant migration of gel filler: imaging findings following breast augmentation. Skeletal Radiol 2022; 51 (11): 2223-2227. doi: 10.1007/s00256-022-04037-1.
